# Association Between Serum Phosphorus and 28-Day Mortality in Patients with Bloodstream Infection: Potential Prognostic Implication Beyond Renal Function and Clinical Severity

**DOI:** 10.3390/pathogens15050553

**Published:** 2026-05-20

**Authors:** Ningjing Pu, Juan Xiong, Yueshan Sun, Ke Li, Yuanbiao Guo

**Affiliations:** 1 Department of Medical Laboratory, The Affiliated Hospital, Southwest Medical University, Luzhou 646000, China; bytgzy@163.com (N.P.); 18782982604@163.com (J.X.); 2 Clinical Laboratory Department, The Third People’s Hospital of Chengdu, The Affiliated Hospital of Southwest Jiaotong University, Chengdu 610031, China; 3 Department of Medical Laboratory, Chengdu Qingbaijiang District People’s Hospital, Chengdu 610300, China; 4 Medical Research Center, The Third People’s Hospital of Chengdu, The Affiliated Hospital of Southwest Jiaotong University, Chengdu 610031, China

**Keywords:** bloodstream infection, hyperphosphatemia, 28-day mortality, risk threshold, metabolic collapse, non-renal phosphorus efflux

## Abstract

Objective: Our objective was to investigate the association between serum phosphorus levels and 28-day mortality in patients with bloodstream infection (BSI), and to explore whether this association persists after adjusting for renal function and clinical severity. Methods: This retrospective cohort study included 214 BSI patients. Patients were divided into hyperphosphatemia (≥2.2 mmol/L, *n* = 15) and control (<2.2 mmol/L, *n* = 199) groups. To address the small sample size and potential separation, multivariate Firth’s penalized likelihood regression was utilized to evaluate the association with 28-day mortality. Restricted cubic spline regression explored the continuous relationship. Fine–Gray competing risk models, 1000-resample bootstrapping, and E-value analyses were conducted to ensure the robustness of the observed associations. Results: The 28-day mortality rate was significantly higher in the hyperphosphatemia group (80.0% vs. 39.7%, *p* = 0.005). After adjusting for age, sex, and estimated glomerular filtration rate (eGFR), hyperphosphatemia remained significantly associated with higher observed 28-day mortality (OR = 4.46, 95% CI: 1.36–18.54, *p* = 0.012). This association remained robust even after further adjustment for septic shock (OR = 4.74, 95% CI: 1.30–21.64, *p* = 0.017). Analyzed continuously, each 0.5 mmol/L increase in serum phosphorus was associated with 34% higher odds of mortality (OR = 1.34, 95% CI: 1.07–1.74, *p* = 0.01). Spline analysis confirmed a nonlinear relationship with a threshold at 2.2 mmol/L. Kaplan–Meier analysis demonstrated a severity-driven survival separation in the hyperphosphatemia group (Log-rank *p* < 0.001). The association remained highly robust after adjusting for early discharge competing risks (sHR = 4.62, *p* < 0.001) and in bootstrap validation (median OR = 4.80). Conclusions: Serum phosphorus ≥ 2.2 mmol/L is associated with higher observed mortality in BSI patients, an association that remained evident after adjusting for renal function and clinical severity, including septic shock. However, given the small hyperphosphatemia subgroup (*n* = 15), limited statistical stability, and the potential for residual confounding, these findings should be considered hypothesis-generating rather than definitive, requiring prospective validation in larger, adequately powered cohorts. Rather than a definitive triage tool, serum phosphorus may serve as a simple, adjunctive marker for early metabolic assessment in severe infections.

## 1. Introduction

Bloodstream infection (BSI) remains a major contributor to intensive care unit mortality, and despite incremental advances in critical care, the early recognition of patients at highest risk continues to pose a clinical challenge [1,2,3]. Widely used markers such as lactate and the qSOFA score, though informative, do not always signal the onset of metabolic collapse in its earliest phase [4,5].

Disturbances in serum phosphorus are common in the setting of severe infection, and a subset of patients with BSI develop hyperphosphatemia [6,7,8]. The conventional view has attributed elevated phosphorus largely to impaired renal excretion [9,10]. More recent work, however, points to an additional mechanism: infection-driven mitochondrial injury and loss of membrane integrity may promote the direct release of intracellular phosphorus into the circulation—a process referred to as non-renal phosphorus efflux [11]. From this perspective, marked hyperphosphatemia may reflect systemic cellular damage rather than serving merely as a proxy for renal dysfunction.

The existing literature on phosphorus as a prognostic marker in BSI has yielded valuable insights, though certain methodological aspects—such as the handling of renal function as a confounder and the potential impact of competing events—warrant further attention [6,7]. In particular, few studies have explicitly addressed the dissociation between phosphorus concentrations and renal function, nor have they adequately accounted for competing events such as early discharge in retrospective designs. Moreover, the handling of small subgroups with extreme outcomes has often been suboptimal, leaving proposed cutoff values on uncertain statistical ground [8].

The present study was undertaken to examine the relationship between serum phosphorus and 28-day mortality in BSI patients using a clinical dataset with temporally aligned laboratory and blood culture data. To mitigate small-sample bias, we employed Firth’s penalized likelihood regression, and we used Fine–Gray models to address competing risks. Restricted cubic spline analysis was also performed to explore potential threshold effects while accounting for renal function and clinical severity. Our aim was to generate more stable estimates of this observed association, and to provide preliminary observations that may inform the non-renal phosphorus efflux hypothesis and contribute to the characterization of metabolic decompensation in BSI.

## 2. Materials and Methods

### 2.1. Study Design and Population

This was a single-center retrospective cohort study approved by the Ethics Committee of our hospital (Approval No. 2025-S-407). In accordance with the Declaration of Helsinki and the retrospective nature of the analysis, written informed consent was waived [12].

The cohort was drawn from patients hospitalized between 1 October 2024 and 1 October 2025. Eligible individuals had positive blood cultures accompanied by clinical signs of bloodstream infection—fever, rigors, or altered mental status—consistent with the Surviving Sepsis Campaign Guidelines [4]. We excluded patients under 18 years of age, those who were pregnant or lactating, and individuals with end-stage renal disease or receiving any form of renal replacement therapy. To minimize iatrogenic confounding, patients who received phosphorus-containing preparations or phosphate binders within the first 24 h of admission were also excluded. Additional exclusions comprised advanced malignancy (Stage IV) with an estimated survival of less than three months, missing data on serum phosphorus, serum creatinine, or 28-day vital status, and the presence of severe liver failure, major trauma, or active autoimmune disease.

Initial screening identified 255 patients with positive blood cultures. After excluding 41 individuals (13 with end-stage renal disease or on dialysis, 7 who had received phosphorus-modifying agents, 7 with advanced cancer, and 14 with incomplete key data), 214 patients remained for analysis. We employed complete-case analysis to avoid biases introduced by imputation [13]. Patients were stratified into two groups according to an exploratory threshold derived from spline analysis and the clinical definition of severe hyperphosphatemia: a control group (serum phosphorus < 2.2 mmol/L, *n* = 199) and a hyperphosphatemia group (serum phosphorus ≥ 2.2 mmol/L, *n* = 15). This cutoff was chosen because the spline curve indicated relatively stable mortality risk below this value, a pattern consistent with earlier observations in critical illness [6,8]; however, the threshold remains exploratory and requires external validation.

### 2.2. Data Collection and Definitions

Data were abstracted from the electronic medical record and included demographics (age, sex) and comorbidities (hypertension, diabetes, chronic kidney disease, and others). To ensure temporal alignment of metabolic and clinical status, all clinical and laboratory parameters were collected within a strict 2 h window of the initial blood culture collection (defined as T0). Recorded variables included vital signs (body temperature, systolic blood pressure, level of consciousness), presence of septic shock, and length of hospital stay. Laboratory data obtained concurrently with the blood culture included complete blood count, inflammatory markers, cardiac biomarkers, liver and renal function tests, electrolytes, serum phosphorus, and lactate; these parameters were selected based on their established prognostic relevance in sepsis [14]. Nutritional risk was evaluated using the Nutritional Risk Score (NRS) [15], and 28-day vital status was recorded as the primary outcome.

Key definitions were as follows. Estimated glomerular filtration rate (eGFR) was calculated using the CKD-EPI 2021 equation [16]. Hyperphosphatemia was defined as serum phosphorus ≥ 2.2 mmol/L [6]. Pathogen classification as Gram-negative or Gram-positive was based on blood culture results [17]. Septic shock was defined according to the Sepsis-3 criteria as the requirement for vasopressors to maintain a mean arterial pressure of ≥65 mmHg and a serum lactate level > 2 mmol/L in the absence of hypovolemia [18]. High nutritional risk was defined as an NRS ≥ 5 [15]. The follow-up period for 28-day mortality began from the time of initial blood culture collection (Day 0).

### 2.3. Statistical Analysis

All statistical computations were performed using R (version 4.3.1, R Foundation for Statistical Computing, Vienna, Austria), with a two-sided *p* value < 0.05 considered statistically significant.

Descriptive statistics and survival analysis. Normally distributed continuous variables are expressed as mean ± standard deviation and compared using the *t*-test; non-normally distributed variables are presented as median with interquartile range and compared using the Mann–Whitney U test. Categorical variables are reported as counts (percentages) and compared with Fisher’s exact test or the chi-square test [19]. Kaplan–Meier curves were constructed to depict 28-day survival, and groups were compared using the log-rank test [20].

Nonlinear and multivariate analyses. Generalized linear models incorporating restricted cubic splines (RCS) were used to examine the continuous relationship between serum phosphorus and 28-day mortality, with a knot placed at 2.2 mmol/L [21]. To avoid overfitting, the model was adjusted solely for eGFR, adhering to the events-per-variable principle (≥10 events per covariate) [22]; a phosphorus level of 1.0 mmol/L served as the reference (OR = 1.0). Multivariate models followed the same principle. Given the number of outcome events, only eGFR was included as a core confounder. Three sequential models were fitted: unadjusted (Model 1), adjusted for age and sex (Model 2), and additionally adjusted for eGFR (Model 3); for Model 3, a complete-case analysis was performed for the 212 patients with available eGFR data. Variance inflation factors were all <5, indicating no substantial multicollinearity [19]. To address small-sample bias and potential complete separation, we applied Firth’s penalized likelihood regression [23]. Firth’s penalized likelihood regression was implemented using the logistf package (version 1.24, R Foundation for Statistical Computing, Vienna, Austria) in R.

Sensitivity and subgroup analyses. Serum phosphorus was analyzed as a continuous variable (per 0.5 mmol/L increase, which approximates the standard deviation of serum phosphorus in this cohort, providing a clinically interpretable unit of change) to reduce optimism bias from data-driven cutpoint selection [24]. Fine–Gray competing risk models were fitted, treating survival with early discharge within 28 days as the competing event, to account for the abbreviated hospital course in the hyperphosphatemia group [25]. Subgroup analyses explored associations by pathogen type, age (<65 vs. ≥65 years), sex, and nutritional risk (NRS < 5 vs. ≥5). Owing to the very small number of hyperphosphatemia patients in the Gram-negative infection subgroup (*n* = 3), formal inference was not performed for this stratum [26,27].

Robustness validation. Stability of effect estimates was assessed with 1000 bootstrap resamples [28]. To evaluate the potential impact of unmeasured confounding, we computed E-values [29]. This study was reported in accordance with the STROBE statement [30].

With the exception of eGFR (missing for two patients), all demographic, comorbidity, and outcome data were complete for the 214 patients included in the analysis. For laboratory variables with occasional missing values, all summary statistics and between-group comparisons were based on available data (available-case analysis); the number of patients contributing to each summary measure is reflected in the degrees of freedom for the corresponding statistical test and was not separately tabulated unless missingness exceeded 5% for a given variable.

## 3. Results

### 3.1. Baseline Characteristics

The final cohort comprised 214 patients with bloodstream infection, of whom 199 (93.0%) had serum phosphorus levels below 2.2 mmol/L and 15 (7.0%) met the criterion for hyperphosphatemia. Baseline demographic features and comorbidity profiles were broadly similar between the two groups (all *p* > 0.05). However, measures of acute illness severity differed substantially, as detailed below and in Table 1.

Patients in the hyperphosphatemia group presented with a markedly more severe clinical picture. Their 28-day mortality was approximately twice that of the control group (80.0% vs. 39.7%, *p* = 0.005). Renal function was substantially worse among hyperphosphatemic individuals, with a median serum creatinine of 199.00 μmol/L (IQR 129.25–457.25) and a median eGFR of 29.77 mL/min/1.73 m^2^ (IQR 12.18–53.55) (eGFR data were available for 212 patients, 99.1%), both significantly different from the control group (*p* = 0.001). Hyperphosphatemic patients also exhibited lower temperatures at the time of blood culture collection, a reduced prevalence of alert consciousness, and higher indices of acute illness severity (all *p* < 0.05). Their hospital course was considerably shorter (median 2 days vs. 17 days, *p* < 0.001), and lactate concentrations were strikingly elevated (median 15.06 mmol/L, IQR 10.50–23.25) relative to controls (median 3.49 mmol/L, IQR 2.39–5.78; *p* < 0.001). Detailed comparisons are provided in Table 1.

### 3.2. Nonlinear Association Between Serum Phosphorus and 28-Day Mortality

**Figure 1:**
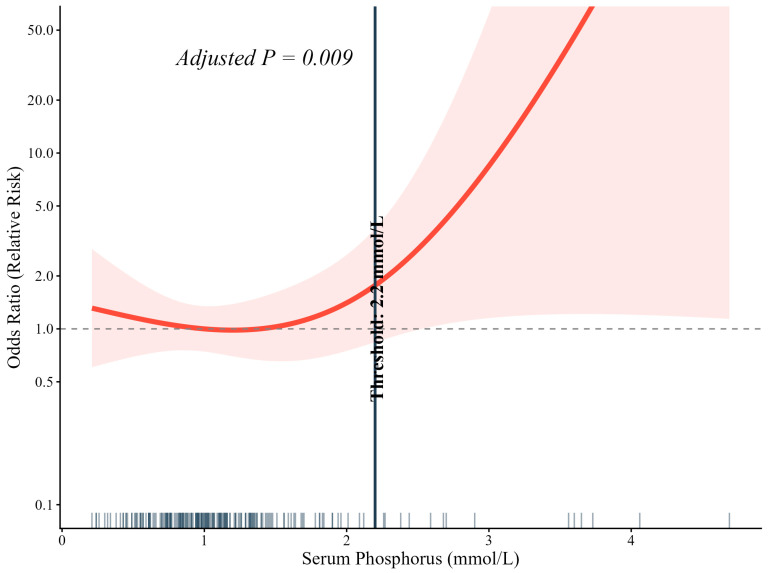
Serum phosphorus and 28-day mortality. With 1.0 mmol/L set as the reference, the risk of death remained relatively flat across lower phosphorus values but rose sharply once levels exceeded approximately 2.2 mmol/L. This inflection suggests a threshold effect and provides empirical support for employing 2.2 mmol/L as an exploratory cutpoint for risk stratification in this cohort. The red line shows the fitted odds ratio, shaded area the 95% CI. Small x-axis ticks show observed value distribution; dashed line denotes OR = 1.0 (reference).

As shown in Figure 1, restricted cubic spline (RCS) regression, adjusted for eGFR, revealed a distinctly nonlinear relationship between serum phosphorus concentration and 28-day mortality (adjusted *p* = 0.009).

### 3.3. Kaplan–Meier Survival Analysis

**Figure 2:**
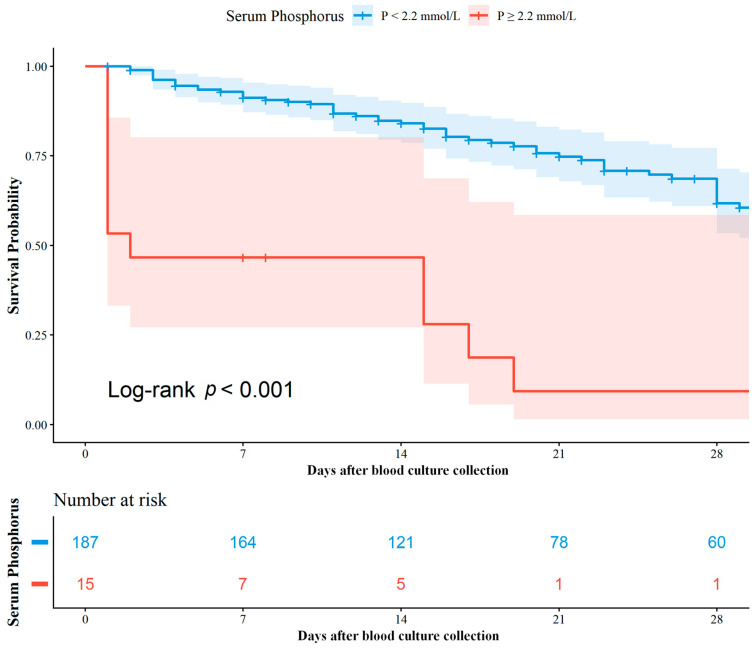
Kaplan–Meier survival curves by serum phosphorus level. The *X*-axis represents follow-up time (days), and the *Y*-axis represents survival probability. The red curve represents the hyperphosphatemia group (*p* ≥ 2.2 mmol/L), and the blue curve represents the control group (*p* < 2.2 mmol/L). The shaded area indicates the 95% confidence interval. The Log-rank test showed a statistically significant difference in survival between the two groups (*p* < 0.001). A number-at-risk table is displayed below the figure, showing the number of patients alive and at risk in both groups at each time point during follow-up.

The Kaplan–Meier curve (Figure 2) showed a rapid early decline in survival in the hyperphosphatemia group. However, given the profound baseline metabolic derangement and hemodynamic instability in this cohort, this early divergence is largely attributable to the greater baseline illness severity in the hyperphosphatemia group, consistent with the higher observed mortality in these critically ill patients. The control group had significantly higher 28-day survival probability (Log-rank *p* < 0.001), indicating that hyperphosphatemia is associated with higher observed mortality in BSI patients.

### 3.4. Multivariate Firth Regression and Sensitivity Analyses

Given that baseline lactate concentrations differed markedly between groups (median 15.06 mmol/L [IQR: 10.50–23.25] vs. 3.49 mmol/L [IQR: 2.39–5.78], *p* < 0.001), indicating profound metabolic derangement, we utilized multivariate Firth’s penalized likelihood regression to assess whether the association between hyperphosphatemia and mortality persisted after accounting for potential confounders. As shown in Table 2, hyperphosphatemia demonstrated a consistent association with 28-day mortality across different adjustment models. After adjusting for age, sex, and renal function (eGFR), hyperphosphatemia remained significantly associated with higher observed 28-day mortality (Model 1: OR = 4.46, 95% CI: 1.36–18.54, *p* = 0.012).

To further evaluate whether this association remains robust after accounting for clinical severity, several sensitivity analyses were performed. The association remained robust even after adjusting for septic shock (Model 4: OR = 4.74, 95% CI: 1.30–21.64, *p* = 0.017). Although the significance was attenuated after further adjustment for qSOFA scores (Model 2: *p* = 0.071) or lactate levels (Model 3: *p* = 0.474), the consistently elevated odds ratios suggest that the association between hyperphosphatemia and mortality is not fully explained by demographic factors and baseline clinical status alone.

We also examined serum phosphorus as a continuous variable. In analyses adjusted for age, sex, and eGFR, each 0.5 mmol/L increment in serum phosphorus was associated with higher odds of 28-day mortality ((OR = 1.34, 95% CI: 1.07–1.74, *p* = 0.01); Supplementary Table S1 and Figure S3). This dose–response pattern suggests that the relationship is stable and not merely an artifact of a single dichotomization threshold.

Given the markedly abbreviated hospital stay observed in the hyperphosphatemia group, we fitted a Fine–Gray competing risk model, treating early discharge as a competing event. The subdistribution hazard ratio for hyperphosphatemia was 4.62 (95% CI: 2.01–10.63, *p* < 0.001), consistent with the primary logistic regression estimate and indicating that the observed association is not substantially driven by differential early discharge (Supplementary Table S2 and Figure S5).

To gauge the internal stability of these estimates, we performed 1000 bootstrap resamples. The median bootstrapped odds ratio for hyperphosphatemia was 4.80 (95% CI: 1.54–30.13), consistent with the primary analysis and supportive of the reproducibility of the findings (Supplementary Figure S1). E-value analysis yielded a value of 8.71 for the point estimate and 2.17 for the lower confidence bound, suggesting that a single unmeasured confounder would need to be moderately strong to fully account for the observed association (Supplementary Figure S2).

Exploratory subgroup analyses were conducted across strata defined by pathogen type, age, sex, and nutritional risk. The microbiological profile of the cohort, including pathogen distribution and antimicrobial resistance patterns, is detailed in Supplementary Table S3. Given the very limited number of hyperphosphatemia cases in the Gram-negative infection subgroup (*n* = 3), formal statistical inference was not undertaken for this stratum.

## 4. Discussion

In this single-center retrospective cohort study, we observed that a serum phosphorus concentration ≥ 2.2 mmol/L was associated with substantially higher 28-day mortality in patients with bloodstream infection. This association remained evident after adjusting for renal function and clinical severity, including septic shock. While the width of the confidence intervals reflects the limited number of hyperphosphatemic cases (*n* = 15), the consistency of the signal across multiple analytic approaches—including continuous variable modeling, Firth’s penalized regression, and competing risk analysis—provides a degree of internal stability to this finding.

This result is consistent with a growing body of evidence across diverse critically ill populations. A prior study found that admission hyperphosphatemia and increasing serum phosphate during hospitalization are independently associated with increased in-hospital mortality [31], and multiple large database studies have replicated this association in both general sepsis and septic shock cohorts [7,32]. By focusing specifically on bloodstream infection and using laboratory parameters obtained concurrently with initial blood cultures (T0), this study provides a temporally aligned assessment that reduces confounding from early resuscitation measures; the observed association persists after adjusting for renal function and clinical severity.

The inflection point identified by restricted cubic spline analysis, approximately 2.2 mmol/L, coincides with the conventional definition of severe hyperphosphatemia. This threshold should be viewed as exploratory, as our continuous variable analysis revealed a dose–response relationship (each 0.5 mmol/L increase was associated with 34% higher odds of mortality; Supplementary Table S1 and Figure S3). The broader literature has examined hyperphosphatemia in various sepsis subpopulations—including patients undergoing continuous renal replacement therapy [33] and those with sepsis-associated liver injury [34]—reinforcing the relevance of phosphorus dysregulation across diverse clinical contexts, though the optimal threshold may vary with case mix [35].

An important dimension not captured by our single-measurement design is the dynamic behavior of serum phosphate. Persistently high phosphate trajectories confer substantially greater mortality risk than transient elevations in sepsis [6], and the concept of “phosphate trajectory stratification” has recently been introduced within a Cardiovascular-Kidney-Metabolic-Sepsis (CKM-Sepsis) framework [36]. Our finding—that a single value ≥2.2 mmol/L identified patients with 80% 28-day mortality—may capture the extreme end of this trajectory spectrum; however, without serial measurements, we cannot determine whether the hyperphosphatemia we observed persisted, rose further, or resolved.

The biological mechanisms linking extreme hyperphosphatemia to adverse outcomes warrant consideration. Impaired renal excretion is a major contributor, yet the persistence of the mortality association after adjustment for eGFR suggests the involvement of additional pathways. One plausible mechanism is non-renal phosphorus efflux, whereby sepsis-induced mitochondrial injury and loss of cellular membrane integrity promote the release of intracellular phosphate into the circulation [11]. Several observations in our data, while descriptive, are consistent with this concept: the hyperphosphatemic patients exhibited markedly elevated lactate, myoglobin, cardiac troponin, and transaminases, consistent with multi-organ cellular injury. Although the mean temperature of 36.40 °C in the hyperphosphatemia group remains above the strict hypothermia cutoff of 36.0 °C, it is consistent with a ‘cold’ phenotype reflecting impaired thermoregulation in the setting of severe circulatory collapse (Table 1). Within the hyperphosphatemic subgroup, serum phosphorus and creatinine were not meaningfully correlated (Spearman r = −0.07; Supplementary Figure S4), hinting at a decoupling of phosphorus from renal indices in the most severely ill. Organ-specific analyses lend additional support: the phosphate-mortality association is particularly pronounced in sepsis-associated liver injury [34], and hyperphosphatemia in the CKM-Sepsis continuum may reflect global cellular energy failure [36]. Direct measurement of circulating markers of cellular necrosis or mitochondrial dysfunction would be required to substantiate this mechanism.

Serum phosphorus is inexpensive, rapidly available, and already integrated into routine laboratory panels. When interpreted alongside lactate and other indices, it may help characterize the depth of metabolic decompensation in severe infection. The concurrent presence of hyperphosphatemia and severe hyperlactatemia identified a subset of patients with particularly high observed mortality, suggesting a potential role as a bedside indicator of profound metabolic crisis; this finding requires confirmation in larger cohorts.

This study has several limitations. The single-center retrospective design and the very small number of hyperphosphatemic patients (*n* = 15) limit generalizability and statistical stability, although Firth’s penalized regression and bootstrap validation (Supplementary Figure S1) support internal stability. Given the attenuation of the association after adjustment for lactate and qSOFA (Table 2, Models 2 and 3), residual confounding by unmeasured or imprecisely measured severity indicators cannot be excluded; while E-value analysis (E-value 8.71 for the point estimate; Supplementary Figure S2) suggests that an unmeasured confounder would need to be moderately strong to fully explain the observed association, the limited precision of these estimates warrants cautious interpretation. Thus, our findings should be interpreted as exploratory and hypothesis-generating rather than definitive. We measured serum phosphorus at only a single time point, precluding trajectory-based analyses [6,36]; the competing risk analysis could not reliably distinguish discharge due to improvement from discharge against medical advice (Supplementary Table S2 and Figure S5); and direct biomarkers of cellular injury were unavailable. Despite these limitations, the consistency of our findings with larger studies supports further investigation. Multicenter prospective studies with serial phosphorus measurements, incorporation of tissue injury biomarkers, and adequately powered pathogen-specific analyses [37] are needed to validate the 2.2 mmol/L threshold and to clarify whether targeted modulation of phosphorus metabolism can improve outcomes in severe infection. Additionally, data on the appropriateness of antimicrobial therapy were not systematically collected, which may confound the relationship between pathogen profile and outcomes.

## 5. Conclusions

In this single-center retrospective cohort, a serum phosphorus concentration ≥ 2.2 mmol/L identified a small group of bloodstream infection patients with substantially higher observed mortality, an association that persisted after adjustment for renal function and clinical severity, including septic shock. The nonlinear risk profile and the consistency of findings across multiple sensitivity analyses are consistent with this observation. However, given the very small hyperphosphatemia subgroup (*n* = 15) and the attenuation of significance after adjustment for lactate and qSOFA, these findings should be considered hypothesis-generating rather than definitive. The concurrent presence of hyperphosphatemia and severe hyperlactatemia provides a phenotypic description that aligns with the concept of non-renal phosphorus efflux and profound metabolic crisis, though mechanistic confirmation remains outstanding. Serum phosphorus may serve as a simple, adjunctive marker for early metabolic assessment in severe infection, but its precise clinical utility and underlying pathophysiology require further prospective evaluation in larger, adequately powered cohorts.

## Figures and Tables

**Table 1:** Baseline characteristics of patients with bloodstream infection.

	P < 2.2 mmol/L	P ≥ 2.2 mmol/L	*p* Value
Demographics & Comorbidities			
*n*	199	15	
Age (mean (SD))	67.52 (15.25)	69.53 (11.29)	0.617
Female, *n* (%)	62 (31.2)	1 (6.7)	0.073
Nutritional_risk (median [IQR])	4.00 [3.00, 5.00]	4.00 [4.00, 6.00]	0.934
Hypertension, *n* (%)	81 (40.7)	8 (53.3)	0.418
Diabetes, *n* (%)	63 (31.7)	8 (53.3)	0.095
Kidney disease, *n* (%)	29 (14.6)	1 (6.7)	0.7
Solid tumor, *n* (%)	38 (19.1)	3 (20.0)	1
Hematological tumor, *n* (%)	8 (4.0)	1 (6.7)	0.487
Vital Signs & Clinical Severity			
Temperature (°C, mean (SD))	37.67 (1.18)	36.40 (0.39)	<0.001
HR (beats/min, mean (SD))	100.27 (23.31)	106.47 (21.30)	0.319
RR (beats/min, mean (SD))	21.34 (5.03)	19.73 (7.18)	0.25
BPS (mmHg, mean (SD))	115.70 (23.79)	94.40 (15.90)	0.001
Consciousness (alert), *n* (%)	113 (56.8)	3 (20.0)	0.007
qSOFA (M [IQR])	1.00 [0.00, 2.00]	2.00 [1.00, 2.00]	0.003
Sepsis, *n* (%)	133 (66.8)	10 (66.7)	1
Septic_shock, *n* (%)	90 (45.2)	10 (66.7)	0.178
Pathogens & Resistance			
*Escherichia coli*, *n* (%)	38 (19.1)	1 (6.7)	0.316
*Klebsiella pneumoniae*, *n* (%)	37 (18.6)	0 (0.0)	0.079
Carbapenem resistant, *n* (%)	22 (11.1)	0 (0.0)	0.375
Laboratory: Blood Routine & Inflammation			
WBC Count (×10^9^/L, M [IQR])	10.05 [6.77, 14.48]	12.80 [11.75, 20.81]	0.003
NEU (×10^9^/L, M [IQR])	8.68 [5.45, 13.21]	10.79 [9.64, 16.94]	0.015
LYM (×10^9^/L, M [IQR])	0.59 [0.30, 0.95]	1.31 [0.71, 2.38]	0.001
NLR (M [IQR])	12.57 [6.71, 28.60]	10.83 [6.13, 19.22]	0.445
PLT (×10^9^/L, M [IQR])	130.00 [69.00, 200.00]	96.00 [64.50, 159.50]	0.264
CRP (mg/L, M [IQR])	87.32 [37.43, 161.00]	66.33 [6.72, 167.44]	0.286
IL6 (pg/mL, M [IQR])	269.00 [58.42, 2296.75]	1399.00 [385.25, 4471.50]	0.041
PCT (ng/mL, M [IQR])	1.56 [0.40, 8.89]	0.84 [0.18, 29.65]	0.835
Laboratory: Cardiac & Coagulation			
MYO (ng/mL, M [IQR])	158.00 [69.00, 515.00]	3000.00 [1325.00, 3000.00]	<0.001
TNT (ng/mL, M [IQR])	55.30 [22.09, 163.00]	272.00 [83.70, 2097.50]	<0.001
CKMB (ng/mL, M [IQR])	2.22 [1.15, 4.61]	8.42 [5.72, 29.80]	<0.001
BNP (pg/mL, M [IQR])	239.70 [98.70, 673.60]	678.70 [194.10, 1500.55]	0.132
LAC (mmol/L, M [IQR])	3.49 [2.39, 5.78]	15.06 [10.50, 23.25]	<0.001
PT (s, M [IQR])	15.70 [14.60, 17.40]	21.10 [18.00, 24.30]	<0.001
APTT (s, M [IQR])	40.95 [36.15, 47.35]	60.50 [45.15, 88.95]	0.001
TT (s, M [IQR])	17.40 [16.30, 18.50]	23.60 [18.70, 32.65]	<0.001
FIB (g/L, M [IQR])	4.58 [3.10, 5.99]	3.47 [1.83, 4.59]	0.013
DDI (mg/L, M [IQR])	2.85 [1.29, 6.89]	14.84 [8.93, 26.66]	<0.001
Laboratory: Hepatorenal & Electrolytes			
eGFR (mL/min/1.73 m^2^, M [IQR]) *	68.51 [38.19, 94.54]	29.77 [12.18, 53.55]	0.001
UREA (mmol/L, M [IQR])	9.95 [6.10, 16.49]	16.07 [11.82, 22.45]	0.012
CREA (μmol/L, M [IQR])	94.00 [69.25, 158.00]	199.00 [129.25, 457.25]	0.001
UA (μmol/L, M [IQR])	273.00 [178.50, 378.75]	518.50 [432.50, 690.25]	<0.001
K (mmol/L, M [IQR])	4.06 [3.60, 4.52]	5.17 [4.66, 6.00]	<0.001
Na (mmol/L, M [IQR])	139.10 [135.85, 143.25]	148.70 [139.10, 152.25]	0.063
Cl (mmol/L, M [IQR])	104.40 [99.30, 109.10]	102.50 [97.50, 105.10]	0.141
Ca (mmol/L, M [IQR])	2.09 [1.95, 2.22]	2.14 [1.81, 2.29]	0.788
Mg (mmol/L, M [IQR])	0.80 [0.70, 0.88]	1.09 [0.96, 1.15]	0.314
Clinical Outcomes			
Icu_admission, *n* (%)	124 (62.3)	14 (93.3)	0.022
Length of hospital stay (d, M [IQR])	17.00 [10.00, 30.00]	2.00 [1.00, 15.00]	<0.001
Mortality28, *n* (%)	79 (39.7)	12 (80.0)	0.005

Notes: * Data for eGFR were available for 212 patients (198 in the *p* < 2.2 mM group and 14 in the *p* > 2.2 mM group). Abbreviations: qSOFA, quick Sequential Organ Failure Assessment; eGFR, estimated glomerular filtration rate; IQR, interquartile range.

**Table 2:** Multivariate Firth’s penalized likelihood regression models for 28-day mortality.

Model	Adjustment Variables	Adjusted OR (95% CI) ^†^	*p* Value
Model 1	Age, Gender, eGFR	4.46 (1.36–18.54)	0.012
Model 2	Model 1 + qSOFA score	3.21 (0.91–14.10)	0.071
Model 3	Model 1 + Lactate	1.71 (0.40–8.53)	0.474
Model 4	Model 1 + Septic shock	4.74 (1.30–21.64)	0.017

Notes: ^†^ Adjusted for the specified clinical variables using Firth’s penalized likelihood logistic regression to provide reliable estimates for the small sample size and sparse event data. All models evaluated the association between hyperphosphatemia (*p* ≥ 2.2 mmol/L) and 28-day mortality. Abbreviations: OR, odds ratio; CI, confidence interval; eGFR, estimated glomerular filtration rate; qSOFA, quick Sequential Organ Failure Assessment.

## Data Availability

The datasets generated and/or analyzed during the current study are not publicly available due to institutional policies regarding patient privacy and strict data protection regulations. However, de-identified data are available from the corresponding authors on reasonable request and subject to ethical approval.

## Supplementary Materials

The following supporting information can be downloaded at: https://www.mdpi.com/article/10.3390/pathogens15050553/s1, Figure S1:Bootstrap Distribution (1000 iterations); Figure S2: E-value Sensitivity Analysis; Figure S3: Restricted cubic spline (RCS) curve for the association between serum phosphorus levels and 28-day mortality; Figure S4: Correlation between serum phosphorus and creatinine in the hyperphosphatemia group; Figure S5: Cumulative incidence functions for 28-day mortality (Fine-Gray model); Table S1: Association between continuous serum phosphorus and 28-day mortality using Firth’s penalized likelihood regression; Table S2: Fine-Gray competing risk model for the association between hyperphosphatemia (≥2.2 mmol/L) and 28-day mortality; Table S3: Microbiological characteristics and resistance profiles of the bloodstream infection cohort stratified by serum phosphorus levels.
