# Supplementary material for: Association Between Serum Phosphorus and 28-Day Mortality in Patients with Bloodstream Infection: Potential Prognostic Implication Beyond Renal Function and Clinical Severity
Source: Pathogens. 2026 May 20;15(5):553. doi: 10.3390/pathogens15050553 (PMC13209206; doi:10.3390/pathogens15050553)
Supplement: Supplementary file 1 [file pathogens-15-00553-s001.zip › Supplementary materials/Supplementary Figures S1-S5.pdf]

Figure S1: Bootstrap Distribution (1000 iterations)

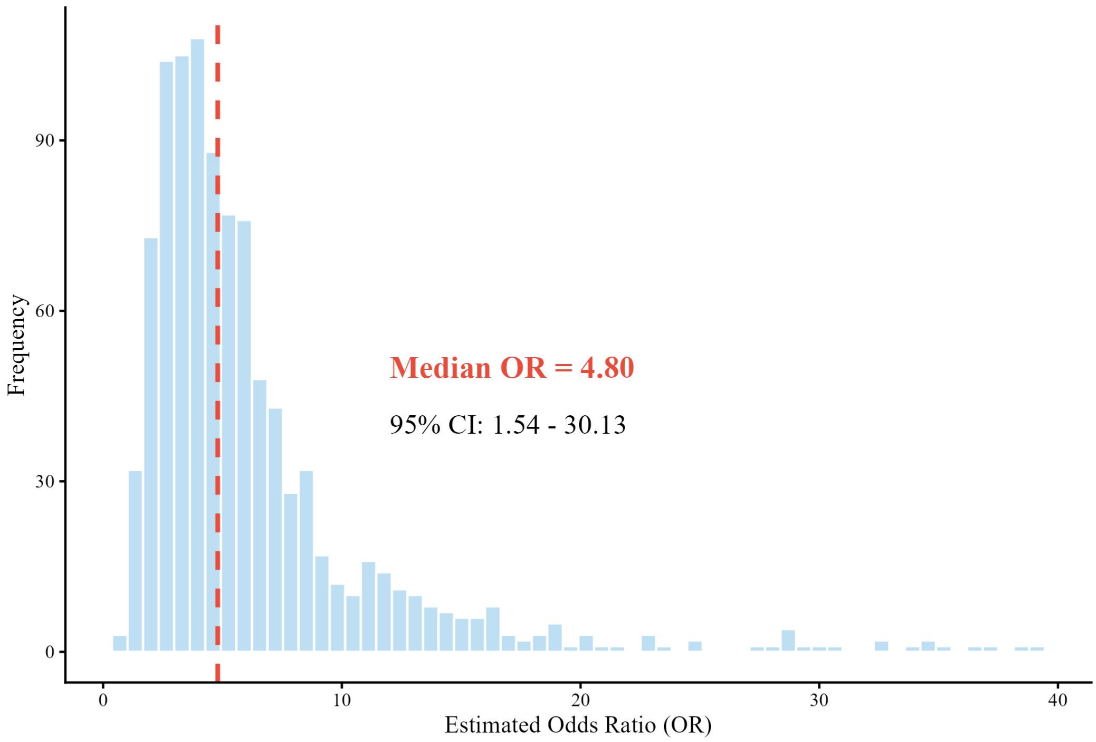

Legend: To verify the stability of the association between serum phosphorus and 28-day mortality, we performed bootstrap resampling (N=1,000). This figure shows the distribution of the ORs obtained from these resamples. The concentration of OR values above 1.0 confirms the robustness of hyperphosphatemia as a consistent risk factor for poor prognosis.

Figure S2: E-value Sensitivity Analysis

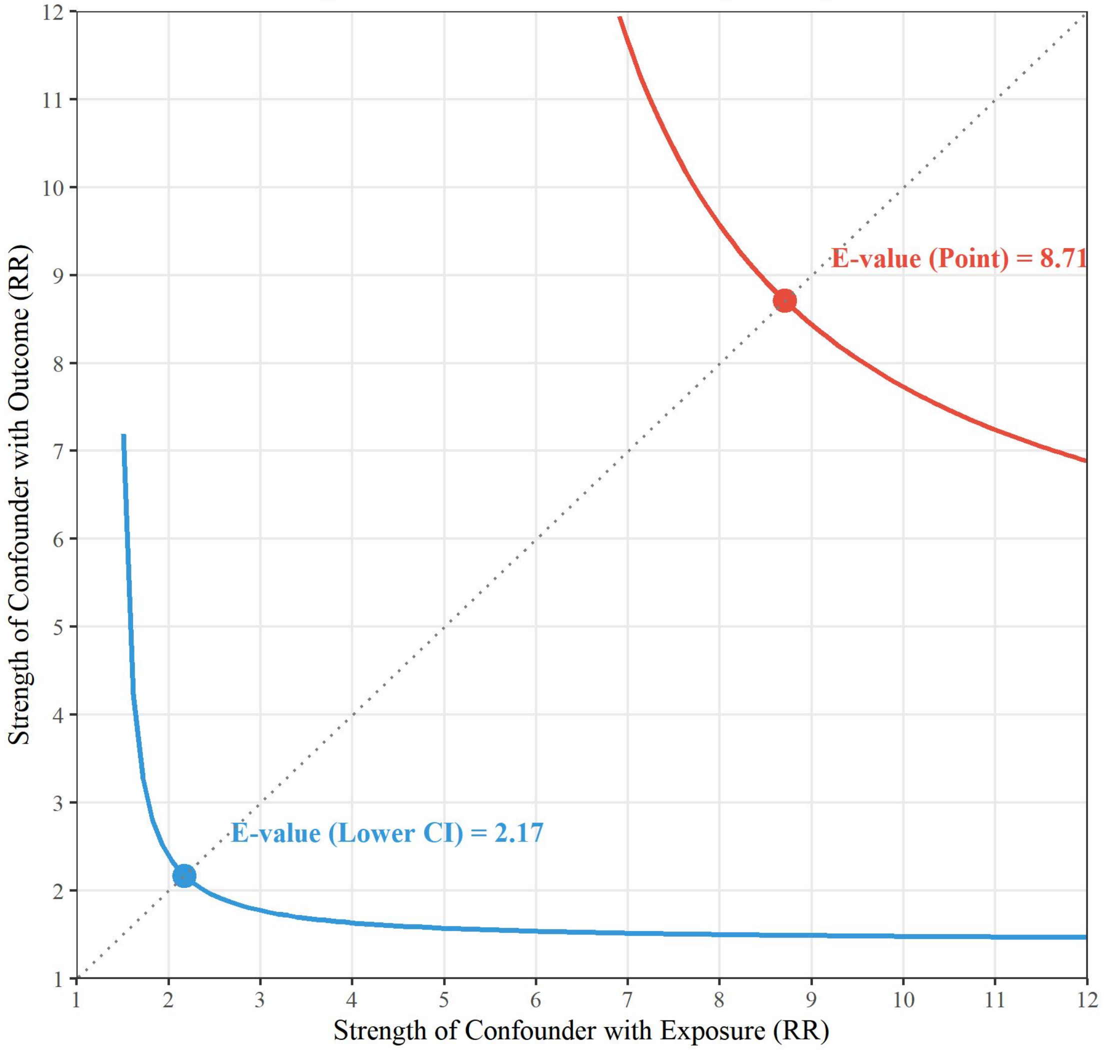

Legend:The E-value was used to assess the impact of unmeasured potential confounding factors on the study results.The results showed that the E-value for the point estimate was 8.71,and the E-value for the lower limit of the confidence interval was 2.17.This means that an unmeasured confounding factor would need to have a relative risk association of more than 8.71 with both blood phosphorus levels and 28-day mortality to counteract the observed effect.This indicates that the results of this study are robust against unmeasured confounders.

Supplementary Figure S3. Restricted cubic spline (RCS) curve for the association between serum phosphorus levels and 28-day mortality.

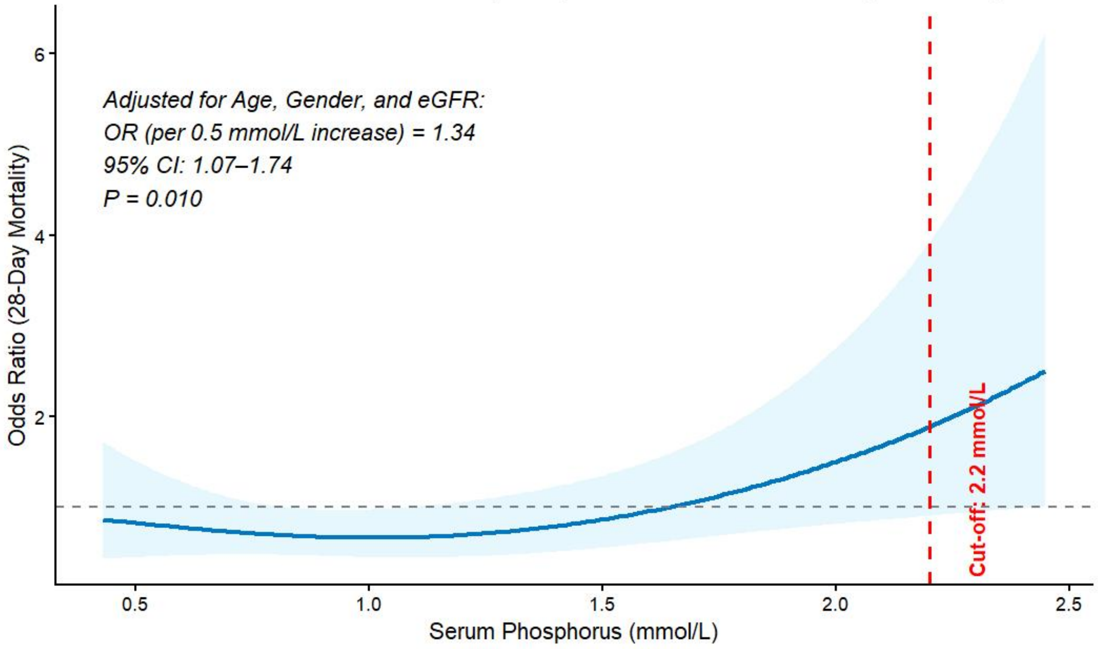

Legend: The dose-response relationship at T0 was modeled using a restricted cubic spline, adjusted for age,gender,and eGFR.The curve illustrates a continuous increase in mortality risk as phosphorus levels rise,with a significant escalation observed beyond the 2.2 mmol/L threshold (red dashed line).

Supplementary Figure S4. Correlation between serum phosphorus and creatinine in the hyperphosphatemia group.

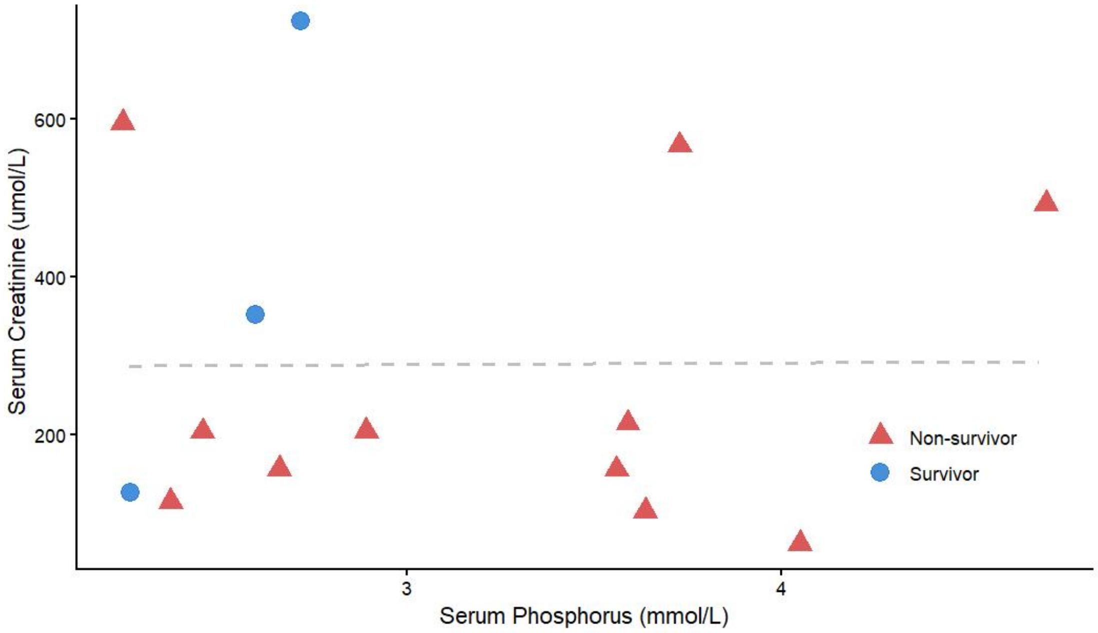

Legend: Among the 15 patients with hyperphosphatemia (≥2.2 mmol/L), the 28-day mortality rate was 80.0% (12/15). The scatter plot displays 14 patients with complete

data (11 non-survivors, red triangles; 3 survivors, blue circles); one non-survivor was excluded due to missing creatinine. The overall linear trend (grey dashed line) demonstrates that high mortality risk persists across a wide range of creatinine levels (60–720 u mol/L), suggesting that the high mortality risk persists regardless of the severity of renal dysfunction.

Supplementary Figure S5. Cumulative incidence functions for 28-day mortality (Fine-Gray model)

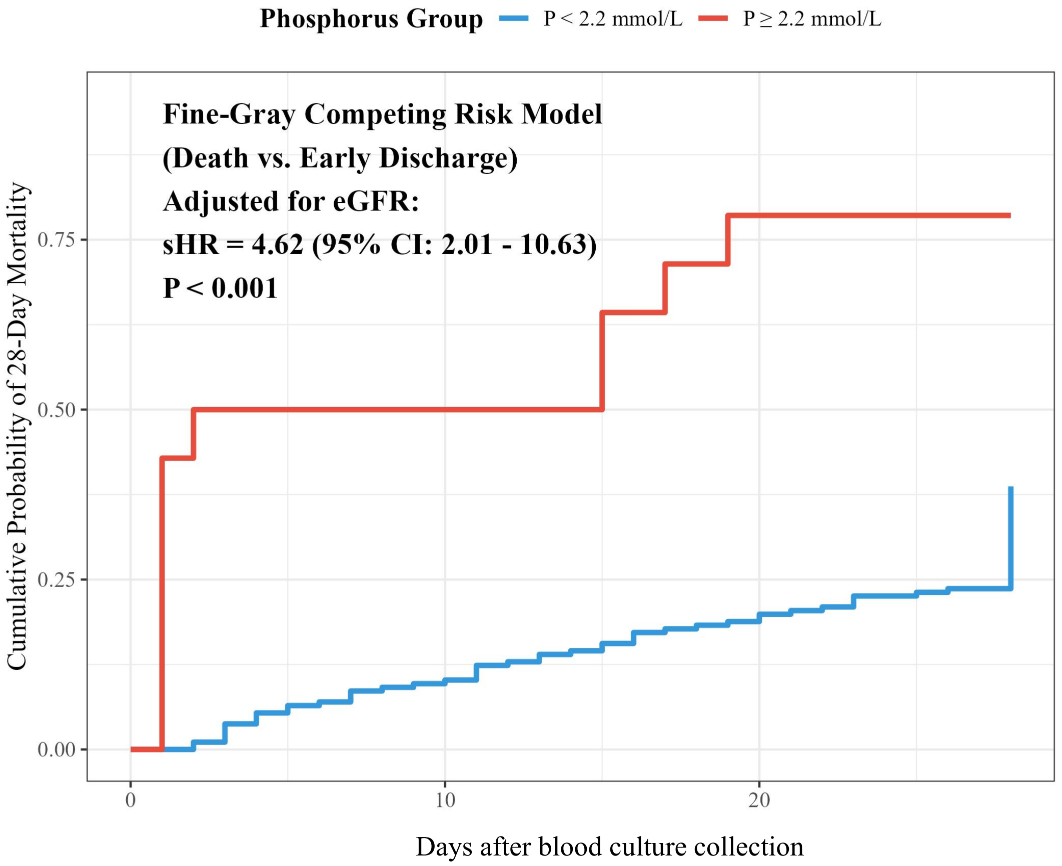

Legend: To account for discharge as a competing risk for in-hospital death, a Fine-Gray model was applied (adjusted for eGFR). Patients with hyperphosphatemia (⩾ 2.2 mmol/L) at T0 showed a significantly higher cumulative incidence of mortality (sHR = 4.62, $P < 0.001$), confirming the consistent prognostic value of serum phosphorus even after accounting for competing events.
